# 规范和提升我国慢性淋巴细胞白血病诊疗能力

**DOI:** 10.3760/cma.j.issn.0253-2727.2023.04.001

**Published:** 2023-04

**Authors:** 录贵 邱, 建勇 李

**Affiliations:** 1 中国医学科学院血液病医院（中国医学科学院血液学研究所），实验血液学国家重点实验室，国家血液系统疾病临床医学研究中心，细胞生态海河实验室，天津 300020 State Key Laboratory of Experimental Hematology, National Clinical Research Center for Blood Diseases, Haihe Laboratory of Cell Ecosystem, Institute of Hematology & Blood Diseases Hospital, Chinese Academy of Medical Sciences & Peking Union Medical College, Tianjin 300020, China; 2 天津医学健康研究院，天津 301600 Tianjin Institutes of Health Science, Tianjin 301600, China; 3 南京医科大学第一附属医院、江苏省人民医院，南京 210029 The First Affiliated Hospital of Nanjing Medical University, Jiangsu Province Hospital, Nanjing 210029, China

随着我国老龄化进程的加快，慢性淋巴细胞白血病（CLL）/小淋巴细胞淋巴瘤（SLL）的发病率不断上升，给CLL/SLL患者的诊断和治疗带来了挑战。为了规范CLL/SLL患者的诊疗，国内外权威学术团体发布了一系列指南[1]。但目前各级医院的临床医师对临床指南的认知和执行情况尚不明确。2021年，中国抗癌协会血液肿瘤专业委员会慢性淋巴细胞白血病工作组开展了一项全国多中心调查研究，研究对象涵盖全国30个省（直辖市/自治区）647所各级医疗机构的1 000名血液科、肿瘤科或淋巴瘤科临床医师。作为我国首项CLL/SLL诊疗现状的全国多中心调查研究，此研究揭示了国内各级医院和医师对CLL/SLL患者的诊断、预后评估、治疗现状，通过与现有指南和最新研究证据对比，发现目前我国相关临床医师在CLL/SLL患者诊疗过程中的不足，为进一步推进CLL/SLL的规范诊疗提供依据。

CLL/SLL的规范化诊断至关重要，多数患者可通过血常规、外周血细胞形态及流式细胞术完成诊断，骨髓穿刺活检等有助于在CLL/SLL诊断困难时与其他淋巴细胞增殖性疾病进行鉴别，此外，在贫血、血小板减少等情况下，骨髓穿刺活检可以准确预估患者的骨髓增生情况，通过观察患者骨髓造血细胞的分布、定位及数量，可评估患者的造血状况[2]。CLL/SLL需要和套细胞淋巴瘤（MCL）、脾边缘区淋巴瘤（SMZL）和华氏巨球蛋白血症（WM）等B淋巴细胞增殖性疾病相鉴别，需要结合B细胞免疫表型、细胞遗传学异常、基因突变等进行综合判定[3]。

此调研显示，目前各级医师对于CLL/SLL治疗指征的掌握及伴随疾病的评估仍显不足。只有13.7%的医师能够正确掌握CLL/SLL患者的治疗指征，即使是CLL中心医院的医师也仅有22.4%能够完全掌握CLL的治疗指征。因此，全国各级医院的医师需加强培训，对于相对容易忽视的起始治疗更要予以充分重视，只有达到治疗指征的患者才需要治疗，以免患者过度治疗或错过最佳治疗时机。CLL患者往往基础疾病较多，常伴心血管疾病等并发症，且由于免疫功能减退容易引发感染，许多CLL患者的最终死亡原因是与血液系统疾病无关的其他疾病。因此，在之后的工作中必须通过疾病教育活动等形式，提升临床医师对CLL患者基础疾病筛查的重视，鼓励医师在治疗前仔细排查患者的基础疾病或伴随疾病，从而降低患者并发症风险，提高治疗的有效率。

在预后评估方面，CLL国际预后指数（CLL-IPI）评分系统是较为有效的工具[4]。但在实际临床工作中，绝大多数医师未能对患者进行CLL-IPI评分，这将不利于医师对患者的治疗和长期管理。此外，在CLL/SLL高危因素的认知上，医师对于免疫球蛋白重链可变区（IGHV）未突变和11q缺失这两个预后高危因素的掌握情况较差。因此，医师需进一步提升对CLL/SLL预后高危因素的认知和关注，从而选择合适的治疗方案，为患者带来长期生存获益。

目前，CLL仍为一种难以治愈的疾病。早期使用烷化剂、嘌呤类似物作为主要的治疗手段。随着CD20单克隆抗体的出现，以利妥昔单抗为基础的免疫化疗时代开始了。然而，传统的FCR（利妥昔单抗联合氟达拉滨、环磷酰胺）、BR（利妥昔单抗联合苯达莫斯汀）方案在老年或伴随严重疾病的患者中不能耐受，在高危、复发难治（R/R）患者中疗效欠佳。近年来，随着对CLL发病机制尤其是B细胞受体信号通路的研究不断深入，许多小分子抑制剂及新的单克隆抗体不断开发应用，CLL的治疗也发生了巨大改变。BTK抑制剂已被多项临床试验证实较免疫化疗能够显著改善初治和R/R患者的长期生存[5]，但目前来自我国的相关证据较少，各级医院应增强组织协作，共同开展多中心临床试验。本调研显示目前医师对于BTK抑制剂的优势患者人群认知仍不够清晰，且减量用药和中断用药的发生率较预期高。因此，在未来仍需要进一步加强BTK抑制剂的规范使用和不良反应管理，根据患者的情况制定个体化治疗方案，提高治疗的有效率。

此外，调研还发现血液科和肿瘤科医师在CLL/SLL的高危因素认知、治疗方案选择、BTK抑制剂临床应用等方面的观念和行为也存在差异，与血液科医师接触到的CLL患者更多、相关诊疗经验更丰富的客观情况一致。因此，未来应对各科室医师进行继续教育，进一步加强其对CLL疾病中高危因素的认知度，同时更好地掌握BTK抑制剂的使用规范和不良反应管理。

我国各级医院的血液科和肿瘤科医师对CLL/SLL的掌握情况，包括治疗指征、预后高危因素、治疗方案选择、BTK抑制剂的临床应用和不良反应管理等仍未达到规范化治疗的理想水平。中国抗癌协会血液肿瘤专业委员会于2022年5月更新了《中国慢性淋巴细胞白血病/小淋巴细胞淋巴瘤的诊断与治疗指南（2022年版）》[6]，对CLL/SLL的诊断、鉴别诊断及规范化治疗进行了修订，总结了最新的循证医学证据，该指南适用于面向全国各级医院医师进行CLL/SLL相关培训。目前已开展的指南解读巡讲活动成功推动了相关指南的传播应用。同时，应当考虑不同水平医院和科室的实际需求，设计适宜的培训内容和方式，如三甲医院和基层医院可进行点对点交流，通过案例展示、专家会议等方式持续向基层医院推广CLL/SLL的规范化诊疗内容，逐步提升诊治水平。CLL/SLL诊治相关检查的同质化也是推动规范化的重要方式，拟在中国抗癌协会血液肿瘤专委会成立相关检查标准化小组，对流式细胞术、染色体核型、二代测序、IGHV检测等方面进行规范。将在全国范围内组织多中心临床试验，努力探索更加适应我国国情的前沿研究方案，同时起到互帮互助的作用。

综上所述，目前我国各级医师在CLL/SLL的规范化诊疗方面仍有很多不足，特别是在诊断、治疗指征识别、预后评估、伴随疾病评估及治疗方案选择方面仍与指南有较大差距。因此，在未来的工作中，专业学术团体要充分发挥组织优势，加大对CLL/SLL诊疗指南的宣传、推广和应用，各级医院加强协作，上下联动，优势互补，稳步提高我国CLL/SLL规范化诊治水平，提高患者疗效。

## References

[1] 中华医学会血液学分会白血病淋巴瘤学组, 中国抗癌协会血液肿瘤专业委员会, 中国慢性淋巴细胞白血病工作组 (2018). 中国慢性淋巴细胞白血病/小淋巴细胞淋巴瘤的诊断与治疗指南(2018年版)[J]. 中华血液学杂志.

[2] Hallek M, Cheson BD, Catovsky D (2018). iwCLL guidelines for diagnosis, indications for treatment, response assessment, and supportive management of CLL[J]. Blood.

[3] 中华医学会血液学分会白血病淋巴瘤学组, 中国抗癌协会血液肿瘤专业委员会, 中国慢性淋巴细胞白血病工作组 (2018). B细胞慢性淋巴增殖性疾病诊断与鉴别诊断中国专家共识(2018年版)[J]. 中华血液学杂志.

[4] 朱 华渊, 王 莉, 乔 佳 (2018). CLL-IPI评分系统在中国慢性淋巴细胞白血病患者中的预后评估价值[J]. 中华血液学杂志.

[5] Hallek M, Al-Sawaf O (2021). Chronic lymphocytic leukemia: 2022 update on diagnostic and therapeutic procedures[J]. Am J Hematol.

[6] 中国抗癌协会血液肿瘤专业委员会, 中华医学会血液学分会, 中国慢性淋巴细胞白血病工作组 (2022). 中国慢性淋巴细胞白血病/小淋巴细胞淋巴瘤的诊断与治疗指南(2022年版)[J]. 中华血液学杂志.

